# Drought stress has transgenerational effects on soybean seed germination and seedling vigor

**DOI:** 10.1371/journal.pone.0214977

**Published:** 2019-09-09

**Authors:** Chathurika Wijewardana, K. Raja Reddy, L. Jason Krutz, Wei Gao, Nacer Bellaloui

**Affiliations:** 1 Department of Plant and Soil Sciences, Mississippi State University, Mississippi State, MS, United States of America; 2 Mississippi Water Resources Research Institute, Mississippi State University, Mississippi State, MS, United States of America; 3 USDA UVB Monitoring and Research Program, Natural Resource Ecology Laboratory, and Department of Ecosystem Science and Sustainability, Colorado State University, Fort Collins, CO, United States of America; 4 USDA, Agriculture Research Service, Crop Genetics Research Unit, Stoneville, MS, United States of America; Estacion Experimental del Zaidin, SPAIN

## Abstract

Effects of environmental stressors on the parent may be transmitted to the F1 generation of plants that support global food, oil, and energy production for humans and animals. This study was conducted to determine if the effects of drought stress on parental soybean plants are transmitted to the F1 generation. The germination and seedling vigor of F1 soybean whose maternal parents, Asgrow AG5332 and Progeny P5333RY, were exposed to soil moisture stress, that is, 100, 80, 60, 40, and 20% replacement of evapotranspiration (ET) during reproductive growth, were evaluated under controlled conditions. Pooled over cultivars, effects of soil moisture stress on the parents caused a reduction in the seed germination rate, maximum seed germination, and overall seedling performance in the F1 generation. The effect of soil moisture stress on the parent environment induced seed quality that carried on the F1 generation seed gemination and seedling traits under optimum conditions and further exasperated when exposed to increasing levels of drought stress. Results indicate that seed weight and storage reserve are key factors positively associated with germination traits and seedling growth. Our data confirm that the effects of soil moisture stress on soybean are transferable, causing reduced germination, seedling vigor, and seed quality in the F1 generation. Therefore, optimal water supply during soybean seed formation period may be beneficial for seed producers in terms of optimizing seed quality and vigor characteristics of commodity seed.

## Introduction

Soybean [*Glycine max* (L.) Merr.] is a globally important annual crop, and one of the major export commodities providing oil and protein for both human and animal food. It is the second most planted field crop in the U.S. next to corn [1] and accounts for about 90% of U.S. oilseed production. Hence, soybean production provides substantial input to the economic structure of the United States.

Soil moisture stress causes extensive losses to soybean production annually [2], and losses due to drought stress are projected to increase due to climate change. Climate change models indicate that historical precipitation patterns will change, and drought stress will become more severe in soybean growing regions in the United States [3]. As soil moisture stress episodes are greatly intensified, bigger importance has been directed in recent years to research on the unfavorable effects of soil moisture stress on soybean crop performance and yield [2].

It is well known that variations in environmental conditions such as photoperiod, water, nutrient status, and solar radiation can have an effect on plant growth and development [4]; however, previous studies have reported that some effects of environmental stressors are transmittable and have fitness and phenotype costs on the first set of offspring from parental generation (F1 generation) [5–7]. For instance, Nosalewicz et al. [8] reported that exposing barley (*Hordeum vulgare* (L.)) to drought stress during reproductive stages decreased the shoot: root ratio and the number of thick roots in the F1 generation. Moreover, exposing *Astragalus nitidiflorus* to drought stress increased seed dormancy in the F1 generation [9].

With the understanding that,effects of environmental stressors are transmittable to the F1 generation of plants that we depend on for food and fiber, then when, where, and how commercial seed is produced may be more critical than originally thought. We know that the quantity and quality of seed are reduced when produced in environments that have erratic precipitation patterns, high evapotranspiration demands, and high atmospheric temperatures, and that seed quality affects germination and seedling vigor [6,7,10–12].

Environmental conditions influence seed size, the concentration of stress hormones in the seed, and germination rates [13,14]. Elevated concentrations of stress hormones in the seed may affect the physiology and phenotypic expression through activation of the abscisic acid responsive element-controlled genes [13]. Additionally, epigenetic mechanisms that affect gene activity without changing the underline DNA sequence are transmitted to successive generations leading to phenotypic modifications in the offspring [15,16]. Many studies on *Arabidopsis thaliana* indicate that stress-induced responses are inherited through the plant’s transgenerational stress memory where the offspring adaptation to particular stress is determined by the stress response established by the parent [17,18]. Some studies have also reported that previous exposure to certain stress would help the plant to acquire tolerance or to develop adaptive mechanisms to a same or different kind of stress during the crop cycle or over the generations [19,20].

In this study, we hypothesized that the effects of drought stress are transferable to the F1 generation and that the transferable effects on the F1 generation are exasperated in low osmotic potential environments during seed germination and in low water-limited environments during early seedling growth. The objective of this study was to determine if the effects of drought stress on soybean are transmittable to the F1 generation and alter seed germination and seedling vigor of the progeny in low soil moisture level environments.

## Materials and methods

### Parental and progeny generations

Three experiments were conducted at the Environmental Plant Physiology Laboratory, Mississippi State University, MS, USA during the 2015 through 2017 growing seasons. The first experiment was conducted in the Soil-Plant-Atmosphere-Research (SPAR) chambers in 2015. These SPAR units can precisely control air temperatures and chamber atmospheric CO_2_ concentration at preset set points and near ambient levels of photosynthetically active radiation (PAR). Each SPAR chamber consists of a 1.27 cm thick Plexiglas which allows 97% of the visible solar radiation to pass without spectral variability in absorption. The Plexiglas chamber (2.5 m tall by 2 m long by 1.5 m wide) accommodates aerial plant parts and a steel soil bin (1 m deep by 2 m long by 0.5 m wide) houses the root system. More details of operation and control of the SPAR facility have been described by Reddy et al. [21].

Seed from an indeterminate (the type which continues to develop new leaves even after the floral induction until photosynthate demand by the developing seed causes a termination in the production of vegetative growth) belonging to maturity group V (Asgrow AG5332) and a determinate (the type which ceases vegetative activity at or soon after photoperiod- and temperature-induced floral induction) belonging to maturity group V soybean cultivar (Progeny P5333RY) were sown into 15.2-cm by 30-cm high polyvinylchloride (PVC) pots that contained a 3:1 mixture of a sand: loam (87% sand, 2% clay, and 11% silt). The selection of these two cultivars were based on their popularity among the producers as these were the commonly grown cultivars in US mid-south region. Before imposing drought stress at R1 (beginning bloom), experimental units were maintained at ambient conditions outside the SPAR units. From 41 days after seeding (DAS) to until physiological maturity (126 DAS), the cultivars were placed in separate SPAR units and exposed to 5 different levels of drought stress, 100, 80, 60, 40, and 20% evapotranspiration (ET) replacement (Table 1) [22]. Parental lines were self-fertilized under uniform SPAR conditions and generated 10 distinct F1 genetic lines, each representing a distinct cultivar by drought stress treatment. At harvest, pods were collected, air-dried at room temperature, and seed separated manually for inclusion in a subsequent germination and vigor experiments. The total number of seed was counted using a seed counter (NP5056-Model 850–2, LiCOR Inc., Lincoln, NE, USA). Individual seed weight was determined by dividing the plant seed weight from seed number per plant. Seed size was determined by the weight. Seed size was divided into three main categories: small (< 12 g/100 seed), medium (12–18 g/100 seed), and large size (>18 g/100 seed).

**Table 1:** Environmental variables during the experimental period.

Treatments	Soil moisture, m^3^ m^-3^	Mean Temperature, °C	[CO_2_], μmol mol^-1^	Mean daily VPD, kPa	Mean daily ET, L d^-1^
100% ET	0.15a†	26.09a	410a	3.5a	15.95a
80% ET	0.14b	26.19a	405a	3.3a	13.80b
60% ET	0.13c	26.48a	408a	4.2a	12.79c
40% ET	0.12d	25.64a	412a	4.0a	8.73d
20% ET	0.11e	25.96a	409a	4.2a	6.54e

Treatments based on the percentage of daily evapotranspiration (ET) imposed at 41 d after planting, average soil moisture, mean temperature, chamber CO_2_ concentration, vapor pressure deficits (VPD), and evapotranspiration (ET) during the experimental period for each treatment.

† Soil moisture values are averaged for each treatment from 41 to 126 days after planting. Values within a column with the different letter are significantly different at P<0.05.

### Measurement of seed quality and chemical composition

Seed from the first experiment was evaluated for protein, oil, fatty acids, sugars, and mineral content, as previously described [23]. Briefly, about 25 g of seed from pooled samples from each cultivar under each treatment was ground using a Laboratory Mill 3600 (Perten, Springfield, IL, USA) and the ground material was analyzed for protein, oil, fatty acids, sugar, and minerals. Seed protein and oil were determined using near infrared (NIR) spectroscopy using a diode array feed analyzer AD 7200 (Perten, Springfield, IL) at the USDA-ARS research facility in Stoneville, MS, USA [24]. Perten’s Thermo Galactic Grams PLS IQ software was used to produce calibration equations, and the curves were established according to AOAC methods [25–28]. Analyses of fatty acids were performed on an oil basis [24,28]. Seed sugar content for glucose, sucrose, raffinose, and stachyose was determined using near-infrared reflectance (AD 7200, Perten, Springfield, IL) and analyzed based on a seed dry matter basis [24,28,29]. The concentrations of seed mineral nutrients including nitrogen (N), phosphorus (P), potassium (K), calcium (Ca), magnesium (Mg), zinc (Zn), copper (Cu), boron (B), iron (Fe), and manganese (Mn) were determined at the Soil Testing Laboratory, Mississippi State University, MS, USA using standard procedures [30].

### Seed germination assay for parents and offspring

The second experiment was conducted to examine the transgenerational effects of soil moisture stress on the F1 generation. Seed germination characteristics (maximum seed germination, time to 50% germination, and seed germination rate), seedling fitness, and tolerance to osmotic stress under *in-vitro* using polyethylene glycol (PEG, molecular weight 8000 Sigma-Aldrich Company, St. Louis, MO, USA) to mimic drought stress were measured. Using a previously described procedure, four replications of 100 seed of two parental lines and their F1 lines developed during experiment 1 were exposed to six levels of osmotic stress including 0.0, -0.1, -0.3, -0.5, -0.7, -0.9 [31]. The incubator was set at 25°C, which was the reported optimum temperature for the germination of soybean seed [32]. The plastic trays were vertically stacked inside the incubator and rearranged every 4 h to minimize the potential of small temperature fluctuations. Seed was considered germinated when the radicle length was greater than half the seed length. Counts were discontinued if no seed in a replication germinated for five consecutive days.

### Maternal effects on seedling vigor

The third experiment was conducted to examine the transgenerational effects of soil moisture stress on seed emergence and seedling vigor, by exposing the F1-generated seed to three levels of drought stress, 100%, 66%, and 33% of field capacity. The experiment was conducted using pre-fabricated mini-hoop structures (rain-out shelters). Each structure consisted of a PVC framework with 4 MIL polythene wrapping having the dimensions of 2-m width × 1.5-m height × 5-m length. Seed was sown in PVC pots (15.2-cm diameter by 30.5-m high) that contained a 3:1 mixture of sand: loam (87% sand, 2% clay, and 11% silt). Pots were arranged in a completely randomized design with 4 replications per cultivar organized in 2 rows with twenty-four pots per row. Four seed were sown in each pot but were thinned to one per pot 1 week after emergence. From emergence until 6 DAS, plants were maintained at 100% of FC. Drought stress treatments were imposed at 7 DAS and continued until harvest, 29 DAS. Throughout the experimental period, plants were fertigated with full-strength Hoagland’s nutrient solution [33] delivered through an automated and computer-controlled drip irrigation system. Soil moisture contents were monitored using Decagon soil moisture sensors (5TM Soil Moisture and Temperature Sensor, Decagon Devices, Inc., Pullman, WA) inserted at a depth of 15 cm in five random pots of each treatment. Irrigation amounts were varied based on measured soil moisture levels to achieve the desired soil moisture treatments.

### Measurements

#### Physiological and gas-exchange measurements

At 26 DAS, leaf chlorophyll (Chl) content, epidermal flavonoids (Flav), epidermal anthocyanin (Anth), and nitrogen balance index (NBI) were measured on the uppermost recently fully expanded leaf with a Dualex^®^ Scientific Polyphenols and Chlorophyll Meter (FORCE-A, Orsay, France). This hand-held instrument enabled to assess the level of chlorophylls in the mesophyll, flavonoids, and anthocyanin in the epidermis, and NBI which indicates nitrogen status using the ratio of chlorophyll and flavonoids units of soybean leaves. Gas exchange and chlorophyll fluorescence parameters were measured using the LI-6400 photosynthesis system (LiCOR Inc., Lincoln, NE). Measurements were made on the uppermost recently fully expanded leaf from three plants in each cultivar from each treatment between 10:00 and 12:00 h. While measuring photosynthesis (Pn), the instrument was set at 1500 μmol photon m^-2^ s^-1^ photosynthetically active radiation, daytime temperature 29°C, 410 μmol mol^-1^ CO_2_, and 50% relative humidity. The flow rate through the chamber was adjusted to 350 mol s^-1^. Pn and the fluorescence (Fv/Fm) were recorded as the total coefficient of variation (%CV) reached a value of less than 0.5. The instrument itself calculates stomatal conductance (g_s_), transpiration (E), and electron transport rate (ETR) by considering incoming and outgoing flow rates and leaf area. Intrinsic water-use-efficiency (WUE) and the ratio of internal (C_i_) to external (C_a_) CO_2_ concentration were estimated as the ratio of Pn/Trans and C_i_/C_a_.

#### Growth and biomass components

At harvest, plant height and the number of nodes on the mainstem were recorded. Leaf area was measured using the LI-3100 leaf area meter (LI-COR, Inc., Lincoln, NE). Stems, leaves, and roots were separated from each plant, and total dry weight per plant was calculated by adding the dry weight of different plant components after oven drying at 80°C for 5 days.

#### Root morphology

After separating the stem from the root system of each plant, roots were washed with water on a sieve [34–36]. Washed roots were scanned with a WinRhizo Pro optical scanner (Regent Instruments, Inc., QC, Canada) by floating the individual root system in 5 mm of water in a Plexiglas tray [34,35]. Gray-scale root images were acquired by setting the parameters to high accuracy (resolution 800 × 800 dpi), and the images were analyzed using WinRhizo Pro software (Regent Instruments, Inc.). From the scanned images, seven root parameters, root length, surface area, diameter, volume, number of tips, forks, and crossings, were recorded.

### Data analysis

Analysis of variance (ANOVA) was performed to determine the differences among the cultivars and treatments for the seed quality traits, mineral composition, growth, physiological, and root developmental parameters using SAS 9.2 (SAS Institute, Inc., Cary, NC, USA). Multiple comparison analyses were performed to identify cultivar-specific responses to treatment effects at the *P* = 0.05 level of significance using the *t*-test. Graphical analysis was carried out using Sigma Plot 13.0 (Systat Software Inc., San Jose, CA, USA).

#### Curve fitting procedure

Germination time-course was fitted with a 3-parameter sigmoidal function (Eq 1) using Sigma Plot 13 (Systat Software Inc.):

$$Y=\mathrm{MSG}/\{1+\exp\;\left[‐\frac{t‐t_{50}}{G_{\mathrm{rate}}}]\right\}$$ Eq 1

where Y is the cumulative seed germination percentage, MSG is the maximum seed germination percentage, t_50_ is the time to 50% maximum seed germination, and G_rate_ is the slope of the curve [37].

Maximum seed germination and seed germination rate (SGR) responses to osmotic potential were analyzed using quadratic (Eq 2) and linear (Eq 3) regression functions. Based on the highest coefficient of determination (r^2^), MSG was modeled using a quadratic function, whereas SGR was modeled by a linear function. [37]. These model functions provided regression constants to estimate minimum osmotic potential when seed germination was zero (MSGOP_min_) (Eq 4) and minimum osmotic potential when seed germination rate was zero (SGROP_min_) (Eq 5), correspondingly, for all treatments and replications in both the cultivars [38,39].

$$\mathrm{MSG}=a+\mathrm{bx}+{\mathrm{cx}}^{2}$$ Eq 2

SGR=a+bx Eq 3

$${\mathrm{MSGOP}}_{\min}=‐b+(\sqrt{+b^{2}}‐4\mathrm{ac})/2c$$ Eq 4

$${\mathrm{SGROP}}_{\min}=‐a/b$$ Eq 5

where *x* is the treatment osmotic potential and *a* and *b* are cultivar-specific equation constants generated using regression functions in Sigma Plot 13 (Systat Software Inc.).

The effect of time, osmotic potential, and cultivar on cumulative percent germination were tested using three-way ANOVA, followed by a Tukey multiple comparison tests. The same statistics were applied to analyze the effect of maternal soil moisture stress effects on offspring germination-based parameters (MSG and SGR) and seedling growth. Also, the replicated values of SGROP_min_ and MSGOP_min_, estimated from linear and quadratic functions, were analyzed using the PROC GLM procedure of SAS. Means were compared using Fisher’s protected least significant difference at *P* < 0.05 probability.

The environmental productivity index (EPI) was utilized to quantify the soil moisture stress effects on estimated seed germination-based parameters such as MSG, SGR, SGROP_min_, and MSGOP_min_ [39–43]. To obtain soil moisture stress indices for the above parameters, the values were normalized by dividing estimated values at control treatment (100% ET) by all estimated values. The resultant relative indices ranged from 0 to 1, where, 1 indicates zero stress or the optimum soil moisture, and 0 indicates total stress or severe water deficit. The developed EPI values were tested to understand the difference between the cultivars and the regression analyses were performed on the relationship between derived values and soil moisture. Critical limits for seed germination-based parameters as a function of soil moisture content were calculated as 90% of the control of soil moisture treatment.

## Results

### Experiment 1: Seed number, seed size, and quality

**Fig 1:**
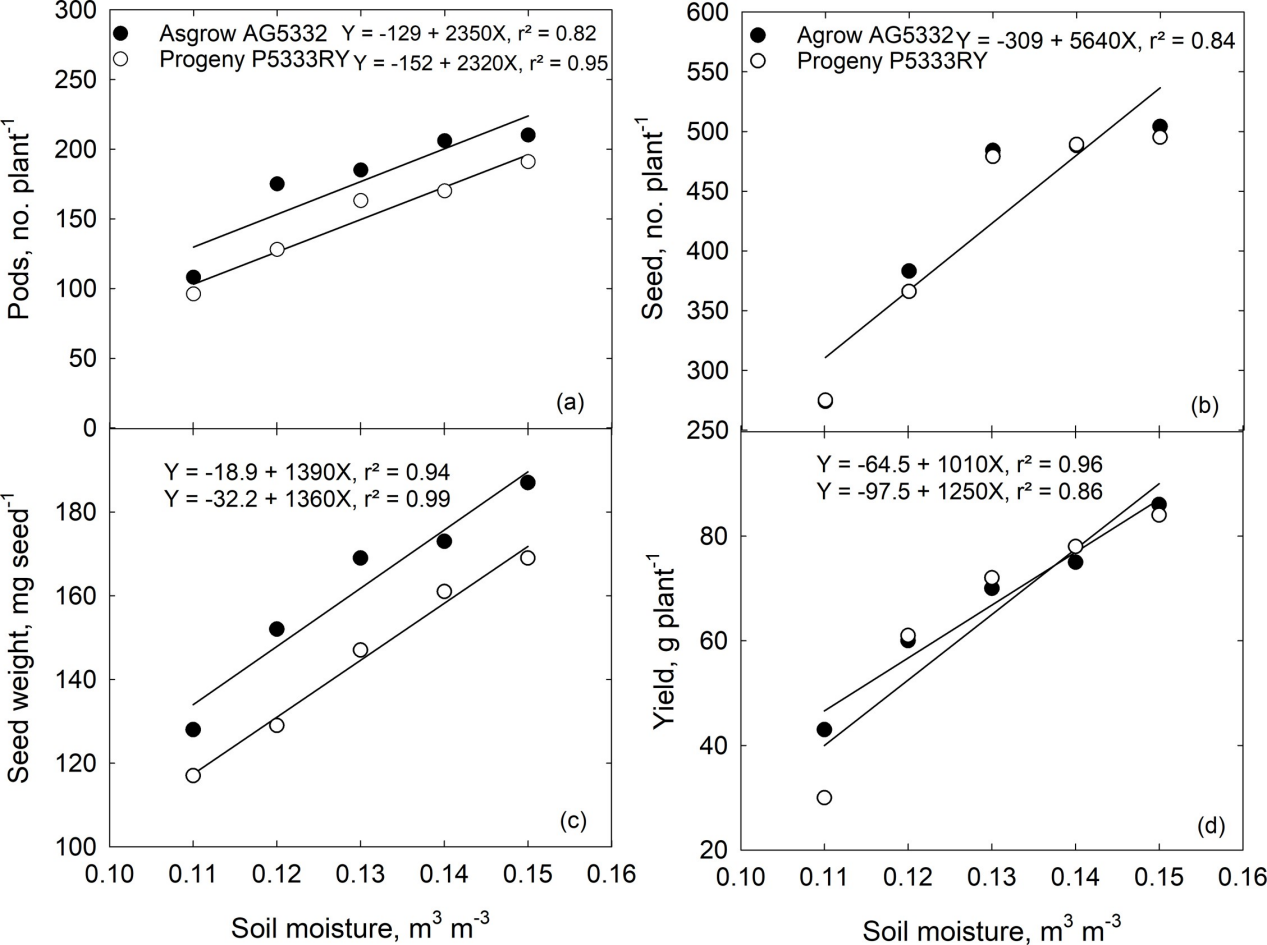
The effect of increasing soil moisture stress (100, 80, 60, 40, and 20% ET) on soybean (a) pod number, (b) seed number, (c) individual seed weight, and (d) yield of Asgrow AG5332 and Progeny P5333RY harvested 126 d after planting.

**Fig 2:**
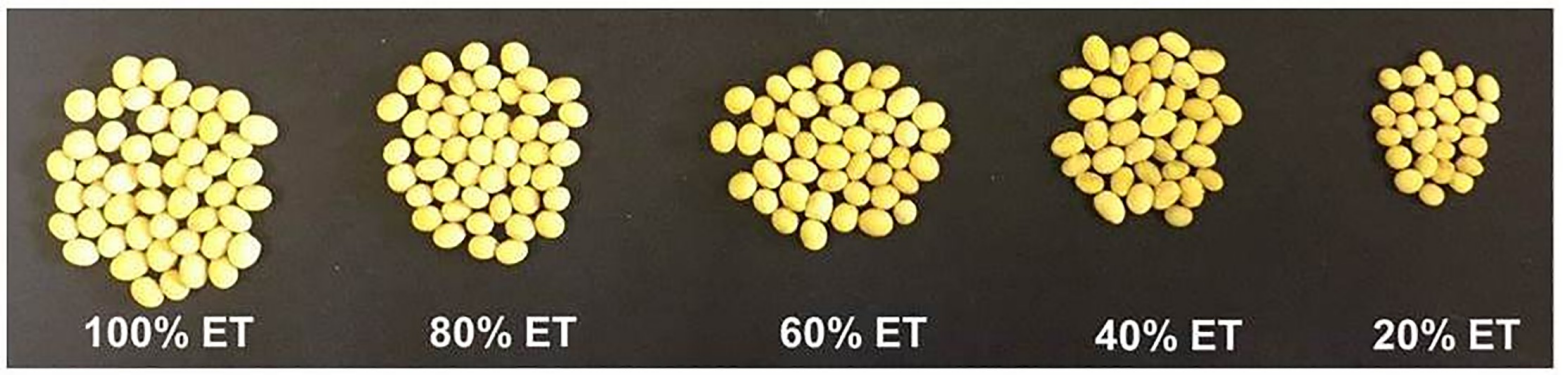
The effect of increasing soil moisture stress (100, 80, 60, 40, and 20% ET) on a soybean seed number, size, and shape of Asgrow AG5332 harvested 126 d after planting. Each seed lot under each treatment represents 1/10 of the total fraction of seed collected from one plant. Due to the absence of significant difference for the seed number between the cultivars, seed only from one soybean cultivar is presented.

Soil moisture stress during reproductive stages had an adverse effect on soybean pod and seed number, individual seed weight, and seed yield (S1 Table, Fig 1). Pooled over cultivar, seed number was positively correlated with soil moisture level. As soil moisture stress intensified, not only did the number of seed decrease, but the production of small, shriveled, and wrinkled seed in the seed lot increased (Fig 2). At severe water deficit (20% ET), most of the seed was long-oval shaped and shrunken, whereas, under well-irrigated condition (100% ET), seed were large, full, and rounded in shape (Fig 2).

The two soybean cultivars showed significant differences (*P*<0.05) for individual seed weight, seed protein, palmitic acid, and nitrogen except sucrose and phosphorus (S1 Fig). Overall, Asgrow AG 5332 showed higher seed weight, proteins, fatty acids, sucrose, and minerals signifying the genetic variation in the sensitivity to the soybean maternal environmental variations. Similar to seed weight, seed protein, palmitic acid, sucrose, nitrogen, and phosphorus also exhibited linear correlations concerning maximum seed germination (S1 Fig).

### Experiment 2: Seed germination traits

#### Cumulative percent germination

For a given osmotic water potential and cultivar, percent germination was inversely correlated with ET replacement level of the parental line. The percent germination of Asgrow AG5332 (S2 Fig) was higher than that of Progeny P5333RY (S3 Fig) at all osmotic potentials except that of -0.9 MPa. No seed from the parental lines of Progeny P5333RY exposed to 40 or 20% ET replacement germinated at an osmotic potential of -0.9 MPa (S3 Fig). No Asgrow AG5332 lines germinated at an osmotic potential of -0.9 MPa (S2 Fig).

#### Maximum seed germination and seed germination rate

**Fig 3:**
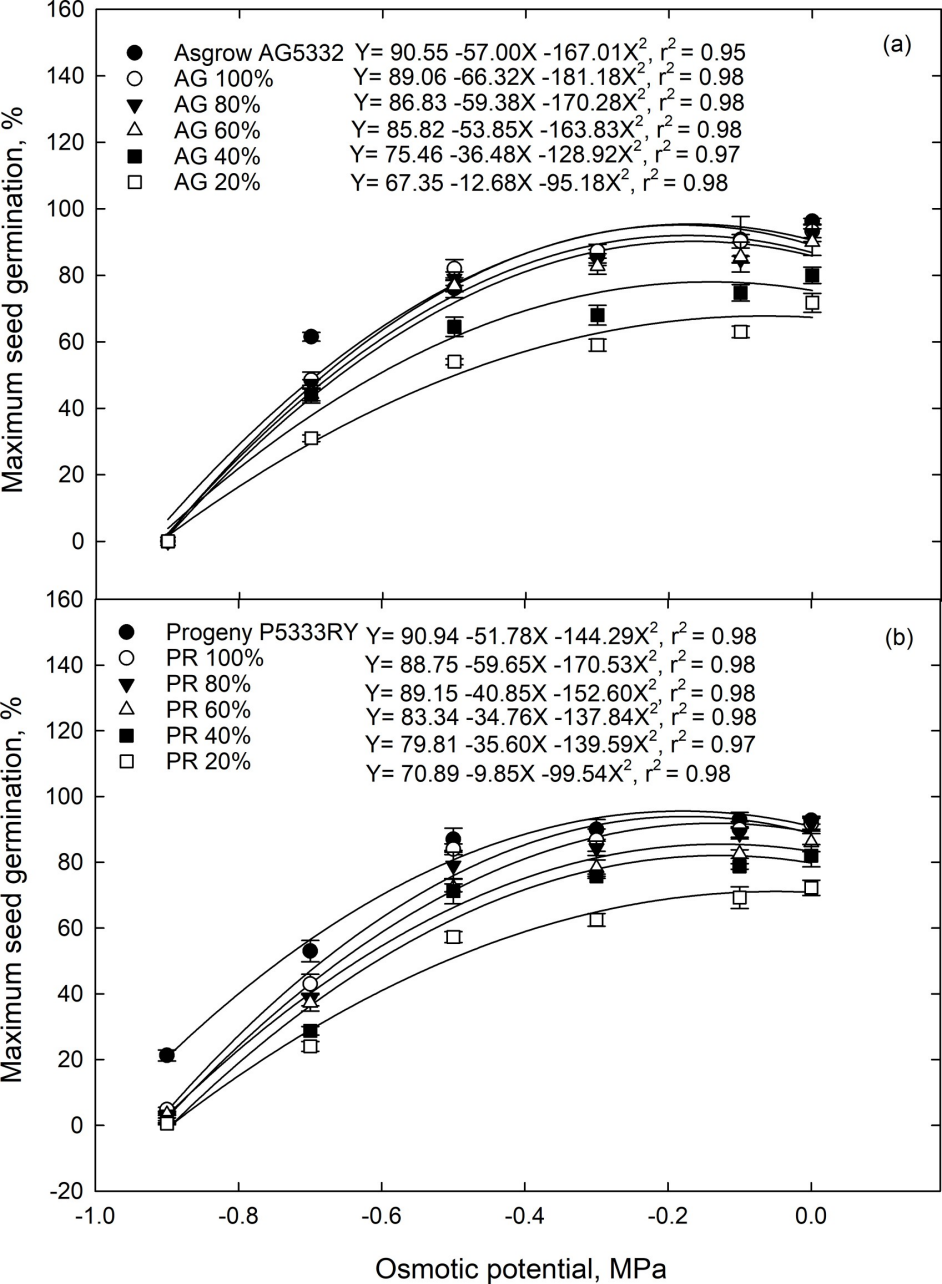
Maximum germination of soybean seed from the F1 generation as a function of osmotic potential. F1 seed was collected from the soybean cultivar (a) Asgrow AG5332 and (b) Progeny P5333RY after exposure to drought stress treatemnts of y including replacement of 100, 80, 60, 40, and 20% of the evapotranspiration demand. Symbols represent the mean of four replications, while bars represent the standard error. Lines are the best fit of a quadratic function.

**Fig 4:**
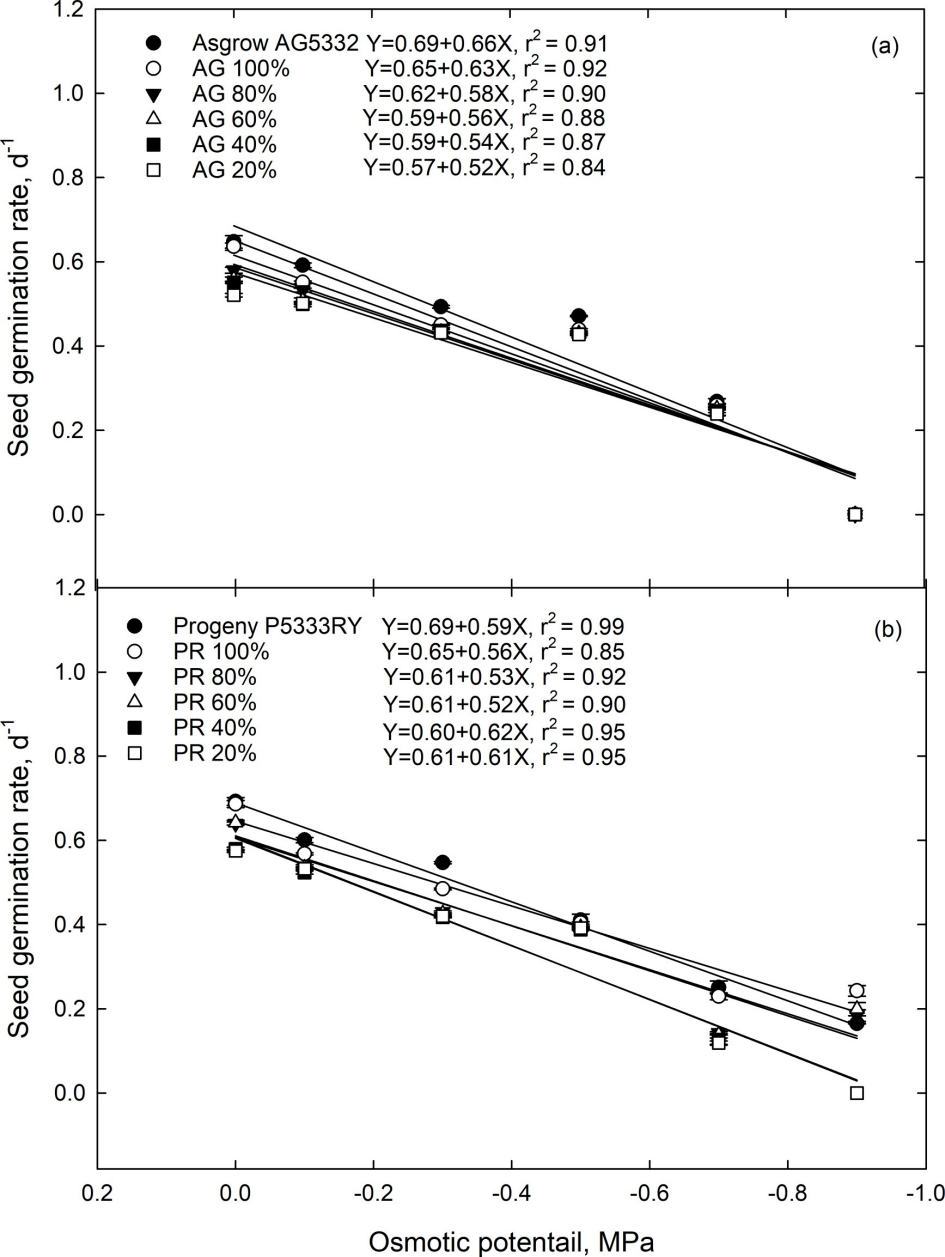
The germination rate of soybean seed from the F1 generation as a function of osmotic potential. F1 seed was collected from the soybean cultivar (a) Asgrow AG5332 and (b) Progeny P5333RY after exposure to drought stress including replacement of 100, 80, 60, 40, and 20% of the evapotranspiration demand. Symbols represent the mean of four replications, while bars represent the standard error. Lines are the best fit of a quadratic function.

Similar to cumulative percent germination, drought stress during the reproductive growth stages of the maternal line caused a decrease in the maximum seed germination and germination rate in the F1 generation (Table 2). Moreover, maximum germination for most osmotic potentials was inversely correlated with parent’s stress level at the time of seed formation (Fig 3). Seed germination rate was correlated with osmotic stress, and the relationship was best described by a linear regression model (mean r^2^ = 0.95) (Fig 4).

**Table 2:** Maximum germination and estimated parameters of soybean seed from the F1 generation as a function of osmotic potential.

Cultivars	MSG (%)	Equation constants			r^2^	MSGOP_min_	Equation constants		r^2^	GRGOP_min_
		a	b	c		(MPa)	a	b		(MPa)
Parent AG	95.40a	90.55	-57.00	-167.01	0.95	-0.93	0.69	0.66	0.91	-1.05
AG 100%	95.13a	89.06	-66.32	-181.18	0.98	-0.91	0.65	0.63	0.92	-1.03
AG 80%	92.01b	86.83	-59.38	-170.28	0.98	-0.91	0.62	0.58	0.90	-1.07
AG 60%	90.25b	85.82	-53.85	-163.83	0.98	-0.91	0.59	0.56	0.88	-1.05
AG 40%	78.04c	75.46	-36.48	-128.92	0.97	-0.92	0.59	0.54	0.87	-1.09
AG 20%	67.77c	67.35	-12.68	-95.18	0.98	-0.91	0.57	0.52	0.84	-1.10
Parent PR	95.00a	90.94	-51.78	-144.29	0.98	-0.99	0.69	0.59	0.99	-1.17
PR 100%	93.97a	88.75	-59.65	-170.53	0.98	-0.92	0.65	0.56	0.85	-1.16
PR 80%	91.88b	89.15	-40.85	-152.60	0.98	-0.91	0.61	0.53	0.92	-1.15
PR 60%	85.52c	83.34	-34.76	-137.84	0.99	-0.91	0.61	0.52	0.90	-1.17
PR 40%	82.08c	79.81	-35.60	-139.59	0.97	-0.89	0.61	0.62	0.95	-0.97
PR 20%	71.13d	70.89	-9.85	-99.54	0.98	-0.89	0.61	0.61	0.95	-1.00

F1 seed was collected from the soybean cultivar Asgrow AG5332 and Progeny P5333RY after exposure to drought stress, including replacement of 100, 80, 60, 40, and 20% of the evapotranspiration demand. Maximum seed germination (MSG), quadratic equation constants (a, b, c), coefficient of determination (r^2^) for MSG, estimated minimum osmotic potential when seed germination was zero (MSGOP_min_), linear regression constants (a, b), coefficient of determination (r^2^) for seed germination rate, and estimated minimum osmotic potential when SGR was zero (SGROP_min_). AG and PG represent Asgrow AG5332 and Progeny P5333RY correspondingly.

The transgenerational effect of drought stress on MSG and SGR differed between cultivars (S2 Table) and was exasperated when the F1 generation was exposed to increased osmotic potentials (Table 2). The seed from 20% ET maternal environment showed the lowest MSG for both Asgrow AG5332 (Fig 3A) and Progeny P5333RY (Fig 3B) cultivars, where they showed 26 and 22% reduction in MSG compared to their parents. The rate of decline was greater for Progeny P5333RY relative to Asgrow AG5332 as osmotic potential increased from 0.0 to 0.9 MPa. There were transgenerational differences in seed germination parameters for cultivar and parental environments (Table 2). At an osmotic potential of 0.0 MPA, the MSG of parent line for Asgrow AG5332 (Fig 3A) was 3.3% greater than that of parent line of Progeny 5333RY (Fig 3B). Stressful maternal environments (20% ET), decreased the rate of seed germination in which it ranged from 0.57 d^-1^ to 0.61 d^-1^ for Asgrow AG5332 (Fig 4A) and Progeny 5333RY (Fig 4B), at 0.0 MPa osmotic potential compared to their parents.

#### Parameter estimates

The two soybean cultivars varied significantly (*P*<0.05) for minimum osmotic potential when MSG was zero (MSGOP_min_) and minimum osmotic potential when SGR was zero (SGROP_min_), which ranged from -0.99 to -0.93 MPa and -1.17 to -1.05 MPa at control condition for Progeny P5333RY and Asgrow AG5332, respectively (Table 2).

The parameters estimated based on EPI (MSG, SGR, MSGOP_min_, and SGROP_min_) declined linearly under moderate and severe water-stressed conditions except for MSGOP_min_ (S4 Fig). Since the two cultivars did not show a significant difference, one linear regression was fitted for the two soybean cultivars. Based on the estimated critical limits (CL), the parameter estimates (MSGOP_min_ CL = 1.116 m^3^ m^-3^ and SGROP_min_ CL = 1.110 m^3^ m^-3^) were lower than MSG and SGR. The critical limit of SGR (0.139 m^3^ m^-3^) was higher than that of MSG (0.133 m^3^ m^-3^).

### Experiment 3: Maternal effects on seedling establishment

**Fig 5:**
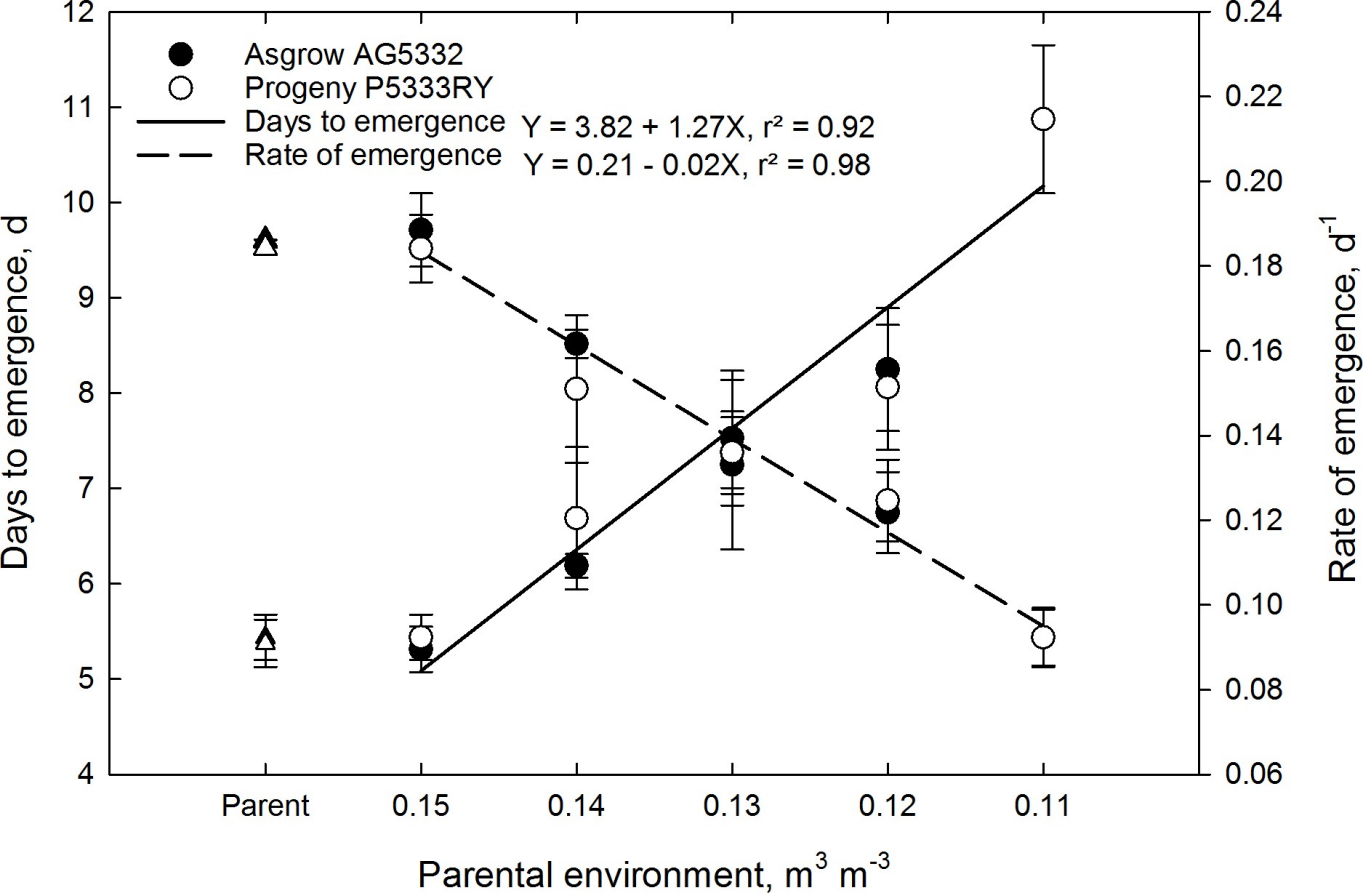
Days to emergence and rate of emergence for the F1 generation of soybean cultivar Asgrow AG5332 and Progeny P5333RY exposed to drought stress including replacement of 100, 80, 60, 40, and 20% of the evapotranspiration demand. Each data point is the mean of four replications pooled over cultivar, while bars represent the standard error of the mean. Lines are the best fit of a linear function.

**Fig 6:**
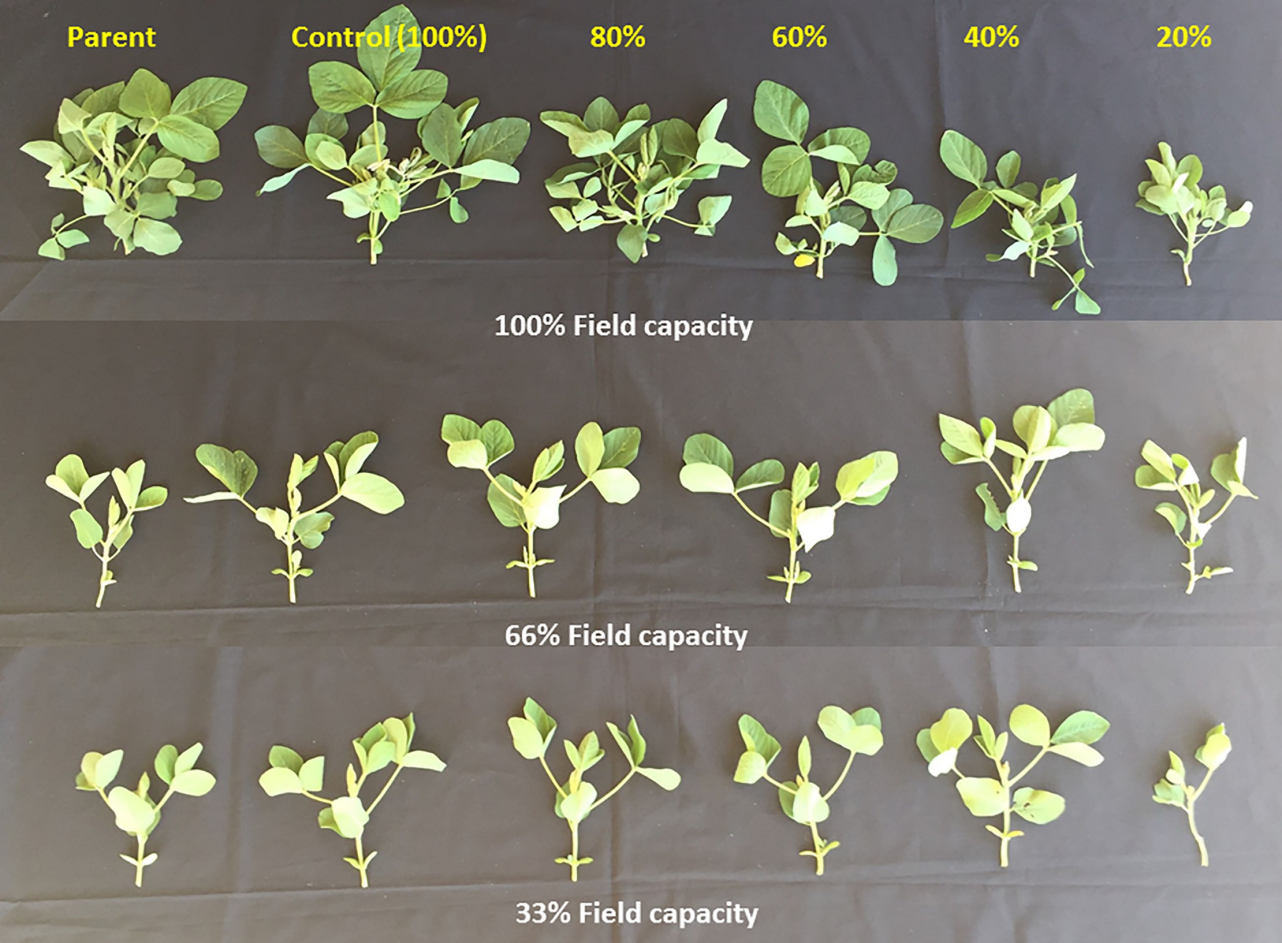
Shoot growth for the F1 generation of soybean cultivar Asgrow AG5332 and Progeny P5333RY exposed to drought stress including replacement of 100, 80, 60, 40, and 20% of the evapotranspiration demand.

The maternal environment affected the rate of emergence of F1 seedlings (Fig 5), and the effect was exasperated (Fig 6) as the average soil moisture content (S5 Fig) decreased from 0.14 to 0.07 m^3^ m^3^. Pooled over cultivar, seed from a maternal environment receiving 100% ET replacement emerged 120% faster than seed from a maternal environment receiving 20% ET replacement (Fig 5).

Drought stress imposed during the reproductive stages of soybean also had a transferable effect on shoot growth and developmental traits (S3 Table). Pooled over cultivar, parameters measured at 29 days after seeding, decreased in response to increasing soil moisture stress for plant height (S6A Fig), leaf area, and biomass components. The leaf area (S6 Fig), stem and leaf dry weights, and total weight (S6 Fig) of the offspring plants grown under both control (100% FC) and 33% FC were consistently lower than the offspring from control maternal treatment (100% ET) and parents. The mean leaf area and total dry weight were reduced by 48% at the 100% FC (control treatment) in Asgrow AG5332 offspring from 20% ET maternal treatment, compared to the parent plant at the same irrigation treatment. The offspring of Progeny P5333RY from 20% ET maternal treatment, on the other hand, exhibited 61 and 55% reduction for leaf area and total dry weight, respectively, compared to its mother plant (S6 Fig).

**Fig 7:**
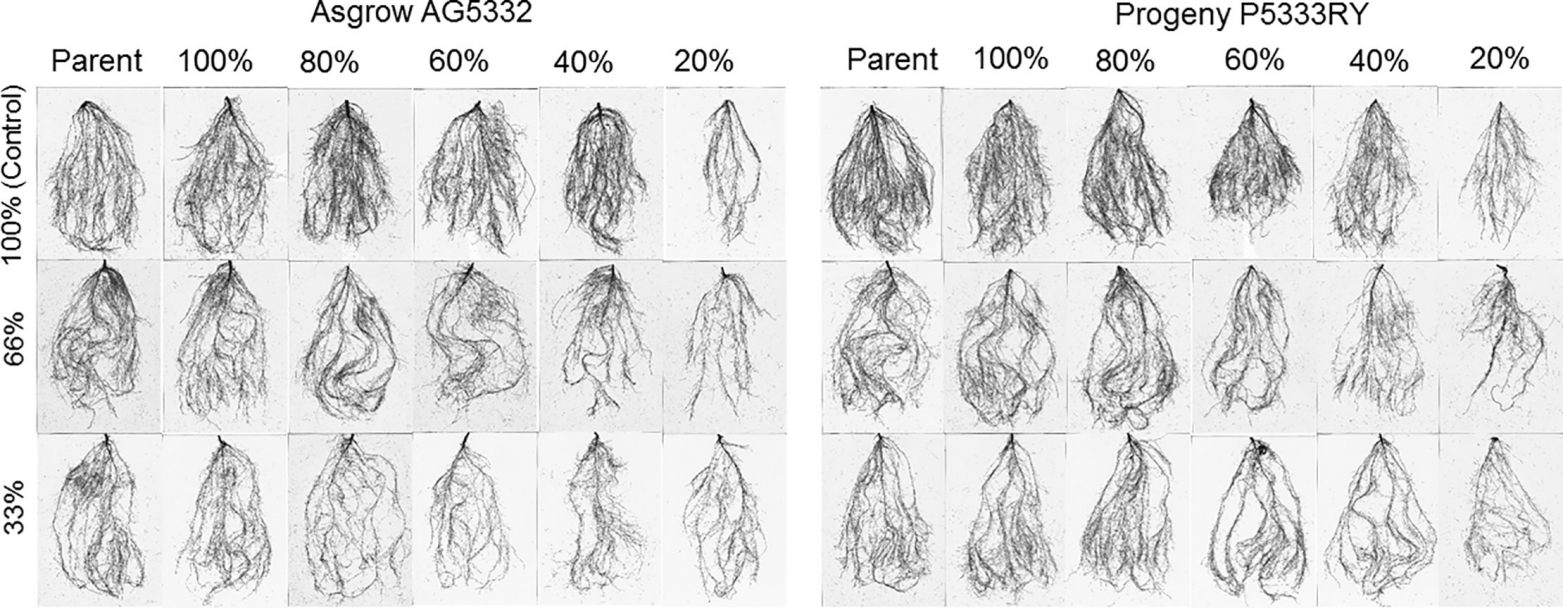
Root growth for the F1 generation of soybean cultivar Asgrow AG5332 and Progeny P5333RY exposed to drought stress, including replacement of 100, 80, 60, 40, and 20% of the evapotranspiration demand.

The number of root tips, forks, and crossings was also lower in soil moisture stressed offspring compared to their parents (S7 Fig). Regardless the maternal environmental effects, all the soybean plants showed less lateral branching close to the surface layer and more taproot elongation towards the deeper layers of soil under sub-optimal soil moisture conditions (66 and 33% FC), compared to the optimum irrigation (100% FC) (Fig 7). Overall, parental plant and the plant from optimum maternal environment had highly branched, longer, and thicker root system compared to the root systems of offspring plants from stressed maternal environments (Fig 7).

Similar to growth and developmental traits, physiological and gas-exchange traits of the offspring were also affected by maternal environments (S3 Table). The lower content of chlorophyll and increased content of flavonoids were observed in offspring from stressed maternal environments (data not presented). The net photosynthesis (Pn), stomatal conductance (g_s_), and electron transport rate (ETR) were consistently lower in the offspring plants than the parents irrespective of the irrigation treatment (S8 Fig). The Pn was reduced by 62, 57, and 53% in Asgrow AG5332 offspring at 100, 66, and 33% FC while the reduction was 48, 57, and 50% for Progeny P5333RY when compared to their parents (S8 Fig). Regardless of the maternal environments, g_s_ and Ci/Ca both decreased with increasing soil moisture stress.

## Discussion

Our data show that during the reproductive stage of soybean, water supply has a pronounced effect on seed quality and seedling vigor of the progeny showing transgenerational consequences. In the present study, soil moisture stress reduced soybean pod and seed number, individual seed weight, and seed yield. The soil moisture stress-induced reduction in seed yield is consistent with the long-known relationship between water supply and yield enhancement during the critical stages of seed development. Other studies also noted that soil moisture stress caused a reduction in seed number and individual seed weight due to the reduction in biomass accumulation and partitioning towards seed when plants were under drought stress [44,45]. When soil moisture stress begins in the early reproductive stages, it causes flower abortion, while drought stress during seed fill reduces carbon assimilation and causes smaller seed, lower seed weights, and reduced seed quality [44].

Seed germination time-course data are important to explain the total viability of seed and lead to estimate the number of seed that will grow into successful seedlings in the field. Moreover, it provides information on the capability of seed to germinate under ideal conditions as well as stress conditions. In the present study, PEG 8000 was used to simulate water stress conditions in germinating the seed. PEG application on the seed in different concentrations has effectively been used in many crop species as a suitable criterion to screen for drought tolerance [33–35]. Our data indicate that the maternal environment has a strong influence on both the timing of germination and viability of soybean seed. Parental seed and the seed from well-irrigated plants in both the cultivars germinated more rapidly than those from unfavorable maternal environments. The percent germination varied substantially for the two cultivars when subjected to the stress of the maternal environment and the proportion of non-germinated seed remained higher at the end of the experiment. Other studies have noted a variation in germination timing in different plant species concerning adverse maternal environments [8,13,46].

When soybean plants were subjected to soil moisture stress at the reproductive stage, the maternal environment significantly affected maximum seed germination and germination rate in both the cultivars. Maximum seed germination decreased as osmotic potential decreased, while seed germination rate decreased linearly in relation to the osmotic stress. The effect of osmotic potential on maximum seed germination and the rate of seed germination could be due to reduced imbibition of water and subsequent effects on embryo growth and development. Seed germination rate is a key factor, which determines seed’s survival potential under optimal or stressful conditions [35]. With rapid germination, the chance of survival is much greater regarding successful stand establishment and rapid resource exploration [33–35,47].

In our study, the parameter estimates, MSGOP_min,_ and SGROP_min_ were obtained from the regression constants derived by the quadratic and linear model functions of MSG and SGR. According to our observations, the parameter estimates were also modified by the maternal environment. Our findings suggest that Asgrow AG5332 exhibits higher osmotic stress at which MSG and SGR become zero or in other words, it has a higher tolerance to drought compared to Progeny P5333RY cultivar at the seed germination stage. These values indicate cultivar’s critical germination potential at the given osmotic stress.

The environmental productivity indices (EPI) concept was used to quantify the effects of soil moisture on maximum seed germination, seed germination rate, and other parameter estimates such as MSGOP_min_ and SGROP_min_ in a changing soil moisture environment in relative terms. Our results indicate that the estimated critical limits for the parameter estimates (MSGOP_min_ and SGROP_min_) were lower than MSG and SGR, indicating their less sensitivity towards soil moisture. The critical limit of SGR was higher than that of MSG; therefore, MSG was more sensitive to soil moisture stress than SGR. This finding suggests that approximately 90% of optimum soil moisture condition, the critical soil moisture level would be 0.11 and 0.14 m^3^ m^-3^ for soybean maximum seed germination and seed germination rate.

The two soybean cultivars showed significant differences in the measured seed quality traits. Overall, Asgrow AG 5332 showed higher seed weight, proteins, fatty acids, sucrose, and minerals signifying the genetic variation in the sensitivity to the soybean maternal environmental variations. Seed size and weight are key characters of determining seed quality and known to be strongly influenced by the maternal environment. Previous studies have found that seed weight was the driving factor on the differences in germination timing due to changes in maternal environments [48]. Agreeing with our observations, individual seed weight linearly correlated with maximum seed germination; with heavier and bigger seed tending to germinate much earlier and faster. Similar to seed weight, seed protein, palmitic acid, sucrose, nitrogen, and phosphorus also exhibited linear correlations with respect to maximum seed germination. This implies that heavier soybean seed with the higher content of seed proteins, fatty acids, sugars, and minerals and storage reserves were able to provide energy more rapidly to germinating seed, which, in turn, increased the maximum seed germination and germination rate. Therefore, the parental environment during seed development apparently could have a significant impact on carbohydrate reserve, proteins, minerals, and overall seed quality.

Although seed weight and quality appeared to affect germination traits for the seed came from stressful maternal environments, some other mechanisms might have involved in the transmission of the observed maternal effects on the progeny. These include epigenetic mechanisms such as histone modifications, DNA methylation, changes in the frequency of homologous recombination, and changes in small and micro RNAs [16,18,19]. Furthermore, direct soil moisture stress effects on the accumulation of metabolites and mRNA or proteins in the seed [6] can also play a vital role in transmitting maternal effects to the offspring. However, our experiment methodology does not permit to differentiate whether the observed changes are due to heritable or non-heritable transgenerational effects, which decreased the fitness of the progeny under the water-stressed conditions similar to their maternal environments.

The aspect of the abiotic maternal environment on the offspring phenotype of the soybean plant has hardly been explored thus far. Therefore, the third experiment was accomplished to evaluate the maternal effects on offspring seedling growth based on their growth, developmental, and physiological characteristics. The maternal environment directly affected the rate of seedling emergence of the offspring. The slower rate of emergence from soybean seed that developed in a 20% ET replacement environment might be due to the effect of drought stress on seed mass and quality [11,45]. The shoot growth and developmental parameters also decreased in response to increasing soil moisture stress. In addition, root length, surface area, root diameter, and the number of tips, forks, and crossings were also lower in soil moisture-stressed offsprings compared to their parents. Regardless of the maternal environmental effects, both the cultivars exhibited an elongated tap root system under sub-optimal soil moisture conditions, compared to the optimum irrigation. Having a longer tap root system could be a drought-adaptive mechanism to increase water and nutrient uptake under stressed conditions [49,50].

Similar to shoot and root traits, physiological and gas-exchange traits of the offspring were also affected by maternal environments. The lower content of chlorophyll and increased content of flavonoids were observed in offspring from stressed maternal environments. Typically, flavonoids production is influenced by genotype and environmental factors [43], particularly an increased production in the leaf as a protective mechanism against abiotic stresses. Regardless of the irrigation treatment, the Pn, g_s_, and Ci/Ca were also lower in the offspring than the maternal plants. Moreover, irrespective of the maternal environments, g_s_, and Ci/Ca both decreased with increasing soil moisture stress. Although many studies have reported that g_s_ is the main factor which is responsible for the net photosynthesis reduction [51,52] compared to non-stomatal limitation such as specific impairments of key metabolic enzymes (Rubisco), decrease in energy consumption, and decrease in the chemical and enzymatic reactions [53,54], our findings suggest the involvement of both stomatal and non-stomatal factors for the decrease in net photosynthesis in soybean plants of the both parents and offsprings. However, in contrary to our findings, beneficial adaptive effects have also been reported in some plants such as faster germination, enhanced resistance, and competitive success in comparison to the offspring of parents not affected by drought stress [55,56]. The possible explanation for these responses would be transgenerational stress memory that allows plants to adapt to future stress events. While we cannot exclude the possibility of such changes in our study, we can at least not confirm the adaptive value of the induced changes.

In conclusion, this research confirms that drought stress during the reproductive growth of soybean affects the fitness and tolerance of the F1 generation to drought stress. The transgenerational effects of drought stress on the germination of the F1 generation were correlated with seed weight, protein, fatty acids, seed carbohydrates, and minerals. The correlation between germination and the seed quality parameters indicate that the drought stress affects how the maternal lines allocate seed storage reserves, which, subsequently, affects seedling emergence and growth in the F1 generation. However, the screening outcomes need to be further validated under field conditions, as crop performance is highly influenced by environmental conditions such as soil, water availability, and temperature.

The effect of the environment has serious ramifications for seed production and multiplication companies. Our data indicate that seed produced in environments where drought occurs during or through the reproductive stages is deleterious to seed production and multiplication. The smaller, poor quality seed that resulted from the stressed maternal environment could result in lower seedling survival in progeny, making them less adapted to withstand drought stress in subsequent generations. However, although we concluded that the main factor responsible for offspring susceptibility to soil moisture stress was the difference in seed size and storage reserve, different epigenetic-based mechanisms also cannot be excluded when describing the maternal effects on offspring fitness.

## Supporting information

S1 Fig **Correlation between maximum germination of soybean seed from the F1 generation of the Asgrow AG5332 and Progeny P5333RY exposed to drought stress including replacement of 100, 50, 60, 40, and 20% of the evapotranspiration demand and (a) seed weight, (b) seed protein content, (c) seed palmitic acid concentration, (d) seed sucrose concentration, (e) seed nitrogen content, and (f) seed phosphorus content.** Soil moisture stress treatments were imposed on the parents at 41 days after seeding, R1 growth stage, and continued until harvest, 126 d after seeding. Symbols represent the mean of four replications. Standard error bars are not displayed when smaller than the symbol for the mean.
(TIF)

S2 Fig Germination of the F1 generation in osmotic potentials ranging from 0.0 to -0.9 MPa. Parent lines for the F1 seed were collected from the soybean cultivar Asgrow AG5332 after exposure to soil moisture stress, including replacement of 100, 80, 60, 40, and 20% of the evapotranspiration demand. Symbols represent the mean of four replications, while bars represent the standard error. Lines are the best fit of the 3-parameter sigmoidal function.
(TIF)

S3 Fig Germination of the F1 generation in osmotic potentials ranging from 0.0 to -0.9 MPa. Parent lines for the F1 seed were collected from the soybean cultivar Progeny P5333RY after exposure to soil moisture stress, including replacement of 100, 80, 60, 40, and 20% of the evapotranspiration demand. Symbols represent the mean of four replications, while bars represent the standard error. Lines are the best fit of the 3-parameter sigmoidal function.
(TIF)

S4 Fig Soil moisture dependent environmental productivity indices (EPI) for maximum seed germination (MSG), seed germination rate (SGR), minimum osmotic potential when MSG was zero (MSGOP_min_), and minimum osmotic potential when SGR was zero (SGROP_min_). Potential values were estimated by dividing the measured parameters by its estimated maximum value at optimum (0.15 m^3^ m^-3^) level and expressed as a fraction between 0 and 1.
(TIF)

S5 Fig Temporal trends in the average soil moisture content for an experiment where the F1 generation of soybean was exposed to three levels of drought stress starting six days after seeding. Drought stress levels included maintaining the soil moisture content at 100, 66, and 33% of field capacity. The F1 generation was collected from the soybean cultivars Asgrow AG5332 and Progeny P5333RY after exposure to drought stress, including replacement of 100, 50, 60, 40, and 20% of the evapotranspiration demand. Symbols represent the mean of eight replications that have been pooled over cultivar. Standard error bars are not displayed when smaller than the symbol for the mean.
(TIF)

S6 Fig Plant height, leaf area, and total dry weight for the F1 generation of soybean cultivar Asgrow AG5332 and Progeny P5333RY exposed to drought stress including replacement of 100, 80, 60, 40, and 20% of the evapotranspiration demand. (TIF)

S7 Fig Root tips, forks, and crossings for the F1 generation of soybean cultivar Asgrow AG5332 and Progeny P5333RY exposed to drought stress including replacement of 100, 80, 60, 40, and 20% of the evapotranspiration demand. Each data point is the mean of four replications pooled over cultivar, while bars represent the standard error of the mean. Lines are the best fit of a linear function.
(TIF)

S8 Fig Photosynthesis, stomatal conductance, and electron transport rate for the F1 generation of soybean cultivar Asgrow AG5332 and Progeny P5333RY exposed to drought stress including replacement of 100, 80, 60, 40, and 20% of the evapotranspiration demand. Each data point is the mean of four replications pooled over cultivar, while bars represent the standard error of the mean. Lines are the best fit of a linear function.
(TIF)

S1 Table Analysis of variance across soybean cultivars, soil moisture stress treatments, and their interactions. Results of the analysis of variance (ANOVA) indicated as *, **, ***, and NS representing significance at the *P* ≤ 0.05, *P* ≤ 0.01, *P* ≤ 0.001, and non-significant (*P* ≥ 0.05), respectively.
(DOCX)

S2 Table Analysis of variance for different seed germination-based parameters based on the parental environment. ***, **, * and NS represent significance level at *P* ≤ 0.001, *P* ≤ 0.05, *P* ≤ 0.01, and *P* > 0.05. Osmotic stress treatments (Trt), parental environment (PE), soybean offspring (Cul), and their interactions (Cul × Trt × PE) with cumulative percent germination (CSG), maximum seed germination (MSG), time to 50% germination (t50), and seed germination rate (SGR).
(DOCX)

S3 Table Analysis of variance across the irrigation treatments (Trt), parental environment (PE), cultivars (Cul), and their interaction (Cul × Trt × PE) with soybean vegetative growth, development, physiological, and root traits measured at 18 days after sowing (DAS). *, **, *** represent Significance levels at *P* ≤ 0.05, *P* ≤ 0.01, and *P* ≤ 0.001. NS represents *P* > 0.05. Nitrogen balance index (NBI), stomatal conductance (g_s_), transpiration (E), the ratio of internal to external CO_2_ concentration (Ci/Ca), fluorescence (Fv/Fm), and electron transport rate (ETR).
(DOCX)

## References

[1] USDA. Periodic and scheduled ERS publications and data on soybeans and oil crops. United States Department of Agriculture Economic Research Service 2017 Accessed on 8/30/2018. https://www.ers.usda.gov/topics/crops/soybeans-oil-crops/#periodic

[2] Leng G, Hall J. Crop yield sensitivity of global major agricultural countries to droughts and the projected changes in the future. Sci. Total Environ. 2019; 654: 811–821. 10.1016/j.scitotenv.2018.10.434 30448671 PMC6341212

[3] Thornton PK, Ericksen PJ, Herrero M, Challinor AJ. Climate variability and vulnerability to climate change: a review. Glob Chang Biol. 2014; 20: 3313–3328. 10.1111/gcb.12581 24668802 PMC4258067

[4] Munnȇ-Bosch S, Alegre L. Cross-stress tolerance and stress ‘memory’ in plants: An integrated view. Environ Exp Bot. 2013; 94: 1–88.

[5] Cendán C, Sampedro L, Zas R. The maternal environment determines the timing of germination in *Pinus pinaster*. Environ Exp Bot. 2013; 94: 66–72.

[6] Figueroa R, Herms DA, Cardina J, Doohan D. Maternal environment effects on common groundsel (*Senecio vulgaris*) seed dormancy. Weed Sci. 2010; 58: 160–166.

[7] Tielborger K, Petru M. An experiment test for effects of the maternal environment on delayed germination. J Ecol. 2010; 98; 1216–1223.

[8] Nosalewicz A, Siecinska J, Smiech M. Transgenerational effects of temporal drought stress on spring barley morphology and functioning. Environ Exp Bot. 2016; 131: 120–127.

[9] Segura F, Vicente MJ, Franco JA, Martinez-Sanchez JJ. Effects of maternal environmental factors on physical dormancy of *Astragalus nitidiflorus* seeds (Fabaceae) a critically endangered species of SE Spain. Flora 2015; 216: 71–76.

[10] Dornbos DL, Mullen RE, Shibles RE. Drought stress effects during seed fill on soybean seed germination and vigor. Crop Sci. 1989; 29: 476–480.

[11] Fahad SA, Bajwa AA, Nazir U, Anjum SA, Farooq A, Zohaib A, et al Crop production under drought and heat stress: plant responses and management options. Front Plant Sci. 2017; 8: 1147 10.3389/fpls.2017.01147 28706531 PMC5489704

[12] Donohue K, Schmitt J. Maternal environmental effects in plants: adaptive plasticity? In: Mousseau T Fox C.W Maternal effects as adaptations (Ed.) Oxford University Press, New York NY 1998 pp 137–158.

[13] Finkelstein RR, Gampala SSL, Rock CD. Abscisic acid signaling in seeds and seedlings. Plant Cell 2002; 14: 15–45. 10.1105/tpc.010441 PMC151246 12045268

[14] Thakur M, Sharma AD. Salt stress-induced proline accumulation in germinating embryos: evidence suggesting a role of proline in seed germination. J Arid Environ. 2005; 62; 517–523.

[15] Lippman Z, Martienssen R. The role of RNA interference in heterochromatic silencing. Nature 2004; 431: 364–370. 10.1038/nature02875 15372044

[16] Berger SL, Kouzarides T, Shiekhattar R, Shilatifard A. An operational definition of epigenetics. Genes Dev. 2009; 23: 781–783. 10.1101/gad.1787609 19339683 PMC3959995

[17] Boyko A, Blevins T, Yao Y, Golubov A, Bilichak A, Ilnytskyy Y, et al Transgenerational Adaptation of *Arabidopsis* to Stress Requires DNA Methylation and the Function of Dicer-Like Proteins. PLoS ONE 2010; 5: e9514 10.1371/journal.pone.0009514 20209086 PMC2831073

[18] Suter L, Widmer A. Environmental heat and salt stress induce transgenerational phenotypic changes in *Arabidopsis thaliana*. PLoS ONE 2013; 8: e60364 10.1371/journal.pone.0060364 23585834 PMC3621951

[19] Jagadish SVK, Muthurajan R, Rang ZW, Malo R, Heuer S, Bennett J, et al Spikelet proteomic response to combined water deficit and heat stress in rice (*Oryza sativa* cv N22). Rice 2011; 4: 1–11.

[20] Paun O, Bateman RM, Fay MF, Hedren M, Civeyrel L, Chase MW. Stable epigenetic effects impact adaptation in allopolyploid orchids (Dactylorhiza: Orchidaceae). Mol Biol Evol. 2010; 27: 2465–2473. 10.1093/molbev/msq150 20551043 PMC2955735

[21] Reddy KR, Hodges HF, Read JJ, McKinion JM, Baker JT, Tarpley L, et al Soil–plant–atmosphere–research (SPAR) facility: A tool for plant research and modelling. Biotronics 2001; 30:27–50.

[22] Wijewardana C, Reddy KR, Alsajri FA, Irby T, Krutz J, Golden B. Quantifying soil moisture deficit effects on soybean yield and yield component distribution patterns. Irrig Sci. 2018; 36: 241–255.

[23] Wijewardana C, Bellaloui N, Reddy KR. Soybean seed physiology quality and chemical composition under soil moisture stress. Food Chem. 2019; 278: 92–100. 10.1016/j.foodchem.2018.11.035 30583452

[24] Wilcox JR, Shibles RM. Interrelationships among seed quality attributes in soybean. Crop Sci. 2001; 41: 11–14.

[25] Association of official analytical chemists (AOAC). Method 988.05 In: official methods of analysis 15th (ed) by Helrich K, Arlington VA. 1990a; AOAC 70.

[26] Association of official analytical chemists (AOAC). Method 920.39 In: official methods of analysis 15th (ed) by Helrich K, Arlington VA. 1990b; AOAC 79.

[27] Bellaloui N, Mengistu A, Fisher DK, Abel CA. Soybean seed composition constituents as affected by drought and *Phomopsisin phomopsis* susceptible and resistant genotypes. J Crop Improv. 2012; 26: 428–453.

[28] Bellaloui N, Smith JR, Ray JD, Gillen AM. Effect of maturity on seed composition in the early soybean production system as measured on near-isogenic soybean lines. Crop Sci. 2009; 49: 608–620.

[29] Boydak E, Alpaslan M, Hayta M, Gercek S, Simsek M. Seed composition of soybeans grown in the Harran region of Turkey as affected by row spacing and irrigation. J Agric Food Chem. 2002; 50: 718–720. 10.1021/jf0255331 12137503

[30] Plank CO. Plant analysis reference procedures for the southern region of the United States Georgia Agriculture Experiment Station Southern Cooperation Service Bulletin 1992; 368.

[31] Michel BE. Evaluation of the water potentials of solutions of polyethylene glycol 8000 both in the absence and presence of other solutes. Plant Physiol. 1983; 72: 66–70. 10.1104/pp.72.1.66 16662983 PMC1066170

[32] Butler TJ, Celen AE, Webb SL, Krstic D, Interrante M. Temperature affects the germination of forage legume seeds. Crop Sci. 2015; 54: 2846–2853.

[33] Hewit EJ. Sand and water culture methods used in the study of plant nutrition Tech. Comm. No. 22. Commonwealth Bureau of Horticulture and Plantation Crops, Commonwealth Agriculture Bureau Farnham Royal, Bucks, England 1952.

[34] Brand D, Wijewardana C, Gao W, Reddy KR. Interactive effects of carbon dioxide low temperature and ultraviolet-B radiation on cotton seedling root and shoot morphology and growth. Front Earth Sci. 2016; 10: 607–620.

[35] Reddy KR, Brand D, Wijewardana C, Gao W. Temperature effects on cotton seedling emergence growth and development. Agron J. 2017; 109: 1379–1387.

[36] Singh B, Norvell E, Wijewardana C, Wallace T, Chastain D, Reddy KR. Assessing morphological characteristics of elite cotton lines from different breeding programmes for low temperature and drought tolerance. J Agron Crop Sci. 2018; 204: 467–476.

[37] Seepaul R, Macoon B, Reddy KR, Baldwin B. Switchgrass (*Panicum virgatum* L.) intraspecific variation and thermotolerance classification using in vitro seed germination assay. Am J Plant Sci. 2011; 2: 134–147.

[38] Singh B, Reddy KR, Redona ED, Walker T. Developing a screening tool for osmotic stress tolerance classification of rice cultivars based on in vitro seed germination. Crop Sci. 2017; 57: 387–394.

[39] Wijewardana C, Alsajri FA, Reddy KR. Soybean seed germination response to in vitro osmotic stress. Seed Technol., 2018; 39: 143–154.

[40] Jones RH, Allen BP, Sharitz RR. Why do early-emerging tree seedlings have survival advantages?: A test using *Acer rubrum* (Aceraceae). Am. J. Bot., 1997; 84: 1714–1718. 21708576

[41] Reddy KR, Hodges HF, McKinion JM. Crop modeling and application: A cotton example. Adv Agron. 1997; 59: 225–290.

[42] Reddy KR, Kakani VG, Hodges HF. Exploring the use of environmental productivity index concept for crop production and modeling In: LR Ahuja V Reddy SA Saseendranand Yu Q ed. Response of crops to limited water: Understanding and modeling of water stress effects on plant growth processes. ASA CSSA SSSA Madison WI 2008; pp 387–410.

[43] Wijewardana C, Reddy KR, Shankle MW, Meyers S, Gao W. Low and high temperature effects on sweetpotato storage root initiation and early transplant establishment. Sci Hort. 2018; 240: 38–48.

[44] Egli DB, Yu ZW. Crop growth rate and seeds per unit area in soybean. Crop Sci. 1991; 31: 439–442.

[45] Samarah NH, Mullen RE, Anderson I. Soluble sugar contents germination and vigor of soybean seeds in response to drought stress. J New Seeds 2009; 10: 63–73.

[46] Donohue K. Completing the cycle: maternal effects as the missing link in plant life histories. Philos Trans B 2009; 364: 1059–1074. 10.1098/rstb.2008.0291 PMC2666684 19324611

[47] Nobel PS. Environmental productivity indices and productivity for *Opuntia ficus indica* under current and elevated atmospheric CO_2_ levels. Plant Cell Environ. 1991; 14: 637–646.

[48] Castro J, Hódar JA, Gómez JM. Seed size In: Basra A.S (Ed.) Handbook of seed science and technology. Haworth Press New York, 2006; pp 397–428.

[49] Fenta BA, Beebe SE, Kunert KJ, Burridge JD, Barlow KM, Lynch PJ, et al Field phenotyping of soybean roots for drought stress tolerance. Agronomy 2014; 4: 418–435.

[50] Kunert KJ, Vorster B, Fenta BA, Kibido T, Dionisio G, Foyer CH. Drought stress responses in soybean roots and nodules. Front Plant Sci. 2016; 7: 1015 10.3389/fpls.2016.01015 27462339 PMC4941547

[51] Ku Y, Wan-Kin A, Yung Y, Li M, Wen C, Liu X, et al Drought stress and tolerance in soybean 2013; InTech 10.5772/52945.

[52] Siddique MRB, Hamid A, Islam MS. Drought stress effects on photosynthetic rate and leaf gas exchange of wheat. Bot Bull Acad Sin Taipei 1999; 40: 141–145.

[53] Cornic G. Drought stress inhibits photosynthesis by decreasing stomatal aperture-not by affecting ATP synthesis. Trends Plant Sci. 2000; 5: 187–188.

[54] Lauer MJ, Boyer JS. Internal CO_2_ measured directly in leaves. Plant Physiol. 1992; 98: 1310–1316. 10.1104/pp.98.4.1310 16668793 PMC1080350

[55] Sultan SE, Barton K, Wilczek AM. Contrasting patterns of transgenerational plasticity in ecologically distinct congeners. Ecology 2009; 90: 1831–1839. 19694132 10.1890/08-1064.1

[56] Walter J, Nagy L, Hein R, Rascher U, Beierkuhnlein C, Willner E, et al Do plants remember drought? Hints towards a drought-memory in grasses. Environ. Exp. Bot. 2011; 71: 34–40.

