# Supplementary material for: Drought stress has transgenerational effects on soybean seed germination and seedling vigor
Source: PLoS One. 2019 Sep 9;14(9):e0214977. doi: 10.1371/journal.pone.0214977 (PMC6733489; doi:10.1371/journal.pone.0214977)
Supplement: S1 Table — Results of the analysis of variance (ANOVA) indicated as *, **, ***, and NS representing significance at the P ≤ 0.05, P ≤ 0.01, P ≤ 0.001, and non-significant (P ≥ 0.05), respectively. (DOCX) [file pone.0214977.s009.docx]

**S1 Table. Analysis of variance across soybean cultivars, soil moisture stress treatments, and their interactions.**

|  | Source | | |
| --- | --- | --- | --- |
| Biomass parameter | Treatment (Trt) | Cultivar (Cul) | Trt × Cul |
| Pod, no. plant^-1^ | *** | *** | NS |
| Seed, no. plant^-1^ | *** | NS | NS |
| Seed weight, mg seed^-1^ | *** | ** | NS |
| Yield, g plant^-1^ | *** | ** | * |

†Results of the analysis of variance (ANOVA) indicated as *, **, ***, and NS representing significance at the *P* ≤ 0.05, *P* ≤ 0.01, *P* ≤ 0.001, and non-significant (*P* ≥ 0.05), respectively.
