# Supplementary material for: Drought stress has transgenerational effects on soybean seed germination and seedling vigor
Source: PLoS One. 2019 Sep 9;14(9):e0214977. doi: 10.1371/journal.pone.0214977 (PMC6733489; doi:10.1371/journal.pone.0214977)
Supplement: S2 Table — ***, **, * and NS represent significance level at P ≤ 0.001, P ≤ 0.05, P ≤ 0.01, and P > 0.05. Osmotic stress treatments (Trt), parental environment (PE), soybean offspring (Cul), and their interactions (Cul × Trt × PE) with cumulative percent germination (CSG), maximum seed germination (MSG), time to 50% germination (t50), and seed germination rate (SGR). (DOCX) [file pone.0214977.s010.docx]

**S2 Table. Analysis of variance for different seed germination-based parameters based on the parental environment.**

| Source | CSG | MSG | t50 | SGR |
| --- | --- | --- | --- | --- |
| Osmotic potential (Trt) | *** | *** | *** | *** |
| PE | *** | *** | *** | *** |
| Offspring (Cul) | *** | *** | ** | ** |
| Trt × PE | *** | *** | *** | *** |
| Cul × PE | *** | ** | ** | ** |
| Trt × Cul | *** | *** | ** | ** |
| Trt × Cul × PE | * | * | * | * |

¶***, **, * and NS represent significance level at *P* ≤ 0.001, *P* ≤ 0.05, *P* ≤ 0.01, and *P* > 0.05. Osmotic stress treatments (Trt), parental environment (PE), soybean offspring (Cul), and their interactions (Cul × Trt × PE) with cumulative percent germination (CSG), maximum seed germination (MSG), time to 50% germination (t50), and seed germination rate (SGR).
