# Supplementary material for: Drought stress has transgenerational effects on soybean seed germination and seedling vigor
Source: PLoS One. 2019 Sep 9;14(9):e0214977. doi: 10.1371/journal.pone.0214977 (PMC6733489; doi:10.1371/journal.pone.0214977)
Supplement: S3 Table — *, **, *** represent Significance levels at P ≤ 0.05, P ≤ 0.01, and P ≤ 0.001. NS represents P > 0.05. Nitrogen balance index (NBI), stomatal conductance (gs), transpiration (E), the ratio of internal to external CO2 concentration (Ci/Ca), fluorescence (Fv/Fm), and electron transport rate (ETR). (DOCX) [file pone.0214977.s011.docx]

**S3 Table.** **Analysis of variance across the irrigation treatments (Trt), parental environment (PE), cultivars (Cul), and their interaction (Cul × Trt × PE) with soybean vegetative growth, development, physiological, and root traits measured at 18 days after sowing (DAS).**

| Source | PE | Treatment (Trt) | Cultivar (Cul) | PE × Cul | Trt × Cul | PE × Trt | PE × Trt × Cul |
| --- | --- | --- | --- | --- | --- | --- | --- |
| Plant parameter |  |  |  |  |  |  |  |
| Seedling emergence, d | * | ** | NS | * | NS | * | * |
| Plant height, cm plant^-1^ | * | * | ** | * | * | ** | * |
| Node no., plant^-1^ | NS | NS | NS | NS | NS | NS | NS |
| Leaf area, cm^2^ plant^-1^ | *** | *** | *** | *** | * | *** | ** |
| Leaf weight, g plant^-1^ | *** | *** | NS | *** | NS | *** | NS |
| Stem weight, g plant^-1^ | *** | *** | NS | *** | NS | *** | NS |
| Root weight, g plant^-1^ | *** | *** | NS | *** | NS | *** | NS |
| Total dry weight, g plant^-1^ | *** | *** | NS | NS | NS | ** | NS |
| Root length, cm plant^-1^ | *** | *** | *** | *** | ** | *** | ** |
| Root surface area, cm^2^ plant^-1^ | *** | *** | NS | *** | NS | *** | NS |
| Root diameter, mm plant^-1^ | * | NS | NS | * | NS | * | NS |
| Root volume, mm^3^ plant^-1^ | *** | *** | NS | *** | NS | *** | NS |
| Root tips no., plant^-1^ | *** | *** | *** | *** | *** | *** | *** |
| Root forks no., plant^-1^ | *** | *** | *** | *** | *** | *** | *** |
| Root crossings no., plant^-1^ | *** | *** | *** | *** | ** | *** | *** |
| Chlorophyll | NS | NS | NS | NS | NS | NS | NS |
| Flavonoids | NS | ** | NS | NS | NS | NS | NS |
| Anthocyanin | NS | NS | NS | NS | NS | NS | NS |
| NBI | NS | * | NS | NS | NS | NS | NS |
| Photosynthesis, µmol CO_2_ m^-2^ s^-1^ | *** | *** | ** | *** | ** | *** | ** |
| gs, mol H_2_O m^-2^ s^-1^ | *** | *** | * | *** | * | *** | * |
| Transpiration, mmol H_2_O m^-2^ s^-1^ | * | * | NS | * | NS | * | NS |
| Fv/Fm | NS | NS | NS | NS | NS | NS | NS |
| ETR, µmol m^-2^ s^-1^ | *** | *** | *** | *** | * | *** | * |
| Ci/Ca | NS | NS | NS | NS | NS | NS | NS |

¶*, **, *** represent Significance levels at P ≤ 0.05, P ≤ 0.01, and P ≤ 0.001. NS represents P > 0.05. Nitrogen balance index (NBI), stomatal conductance (g_s_), transpiration (E), the ratio of internal to external CO_2_ concentration (Ci/Ca), fluorescence (Fv/Fm), and electron transport rate (ETR).
